# Self-testing for cancer: a community survey

**DOI:** 10.1186/1471-2407-8-102

**Published:** 2008-04-14

**Authors:** Sue Wilson, Angela V Ryan, Sheila M Greenfield, Sue C Clifford, Roger L Holder, Helen M Pattison, David A Fitzmaurice, Richard J McManus

**Affiliations:** 1 Department of Primary Care and General Practice, Primary Care Clinical Sciences Building, University of Birmingham, Edgbaston, Birmingham, B15 2TT, UK; 2 School of Life & Health Sciences, Aston University, Aston Triangle, Birmingham, B4 7ET, UK

## Abstract

**Background:**

Cancer-related self-tests are currently available to buy in pharmacies or over the internet, including tests for faecal occult blood, PSA and haematuria. Self-tests have potential benefits (e.g. convenience) but there are also potential harms (e.g. delays in seeking treatment). The extent of cancer-related self-test use in the UK is not known. This study aimed to determine the prevalence of cancer-related self-test use.

**Methods:**

Adults (n = 5,545) in the West Midlands were sent a questionnaire that collected socio-demographic information and data regarding previous and potential future use of 18 different self-tests. Prevalence rates were directly standardised to the England population. The postcode based Index of Multiple Deprivation 2004 was used as a proxy measure of deprivation.

**Results:**

2,925 (54%) usable questionnaires were returned. 1.2% (95% CI 0.83% to 1.66%) of responders reported having used a cancer related self test kit and a further 36% reported that they would consider using one in the future. Logistic regression analyses suggest that increasing age, deprivation category and employment status were associated with cancer-related self-test kit use.

**Conclusion:**

We conclude that one in 100 of the adult population have used a cancer-related self-test kit and over a third would consider using one in the future. Self-test kit use could alter perceptions of risk, cause psychological morbidity and impact on the demand for healthcare.

## Background

Self-tests enable an individual to check for signs of certain health conditions without recourse to a health professional by getting a result immediately e.g. most prostate specific antigen (PSA) and faecal occult blood (FOB) tests, or by sending a sample to a laboratory that returns the result directly to the individual e.g. some chlamydia tests. Such tests can be diagnostic e.g. urine tests for pregnancy, for disease monitoring e.g. blood pressure, or both e.g. PSA tests. Self-tests related to the diagnosis of more than 20 different conditions are available to the UK public via the internet [1].

Several reports have expressed concern about the development of self-testing [2-4]. Potential problems highlighted include: lack of professional support when receiving bad news; lack of expertise to interpret or act on results; unreliable results generating false security or anxiety; that individuals may be forced to take tests by people other than health professionals (e.g. employers); the breakdown of public health surveillance; and that commercially driven test development may lead to demands which the NHS is unable to meet for further testing or treatment. Much of the coverage of self-testing in the press is also negative, warning of the unreliability of tests and the dangers of misunderstanding medical information [5-7].

Despite this, in 2003, market researchers reported that "almost six in ten Britons diagnose themselves at home with self-testing equipment instead of going to the doctor" [8], although this figure does include thermometers as a self-test device. Sales of self-testing equipment are reported to have increased dramatically: almost ₤54.3 m was spent on self-diagnostic products in 2002, a 32% growth since 1998 [9], and it was predicted that would rise to over £60 m by 2007 [8]. Long waits to see a GP and an increasingly health-conscious population are among the factors thought to contribute to the increased sales [10]. Other possible reasons include privacy and convenience.

In the UK, NHS Direct and drop-in health centres aim to increase access to health services and health information [11]. Such initiatives, together with interventions aiming to redefine patients as consumers e.g. Patient Advice and Liaison Services, the increased availability of over the counter medication and funding pressures on health service providers, have encouraged the development of a self-care culture with people taking more responsibility for their own health [12-14]. Self testing may be considered to be a potentially important part of self care within conventional medicine, for example empowering people within a consultation with their doctor, as has been seen with the use of internet resources [15]. Conversely though self-tests may be used by those not wishing to bother doctors, perhaps appealing to those who are dissatisfied with or mistrustful of doctors or conventional medicine.

Health Which [7], the press [5,6] and cancer support groups [16] have reported the availability of cancer self-test kits from high street chemists. Cancer-related self-tests that are currently available in UK pharmacies and over the internet include those for faecal occult blood, PSA and haematuria [1]. Should Internet sales be shown to be profitable, a wider range of cancer self-test kits is likely to become available from pharmacies. Saliva tests for breast cancer risk [17] and kits for testing the response to alternative cancer treatments [18] are already being marketed via the internet. Other potential new developments include a saliva test for breast cancer [19], bladder cancer home tests (currently prescription only) [20], and tests related to genetic determinants of cancer [21] and drug effectiveness [22].

Despite this, little is known about the extent to which people screen themselves for cancer or their reasons: there are no studies that have determined the extent of cancer-related self-test use in the UK. The limited literature that does address self-testing has also tended to concentrate on efficacy and reliability [23,24], has been carried out in different health cultures in the US or Europe [25], or is based on opinion only without empirical data [26]. Self-tests for cancer could alter perceptions of risk and health behaviour, cause psychological morbidity and have a significant impact on the demand for healthcare. Furthermore, they may impact on the cost-effectiveness of population-based screening. It is essential, therefore, that we gain an understanding of the frequency of self-testing for cancer, characteristics of users, and the impact on users and the health service. This study aimed to determine the prevalence of cancer-related self-test use.

## Methods

The methods used are described more fully elsewhere [27]. Following ethical and RM&G approval (Solihull LREC, 22nd March 2005, Ref: 05/Q2706/13, Birmingham and Solihull PCT Research Management and Governance Approval, 29/06/05, Project No: 754), four general practices in Birmingham were recruited. Postal questionnaires and prepaid envelopes were sent to 5,545 people aged over 18 and registered with the participating practices. People that the GP deemed inappropriate to approach, for example because of recent bereavement, were excluded.

A covering letter briefly defined 'self-testing' as "a test or kit bought from a shop or over the internet that is used to see if you have a condition or disease **without **involving a doctor or a nurse", explained the study and requested that a blank form be returned if they did not wish to participate. The three-page questionnaire collected socio-demographic information and data regarding previous and potential future use of self-test kits. The questionnaire was piloted to ensure readability, comprehension, and acceptability. To minimise response bias, questions concerned self-testing for a range of conditions, rather than just cancer, were included. Respondents were asked whether they had used a self test for 18 different conditions (including diabetes, cholesterol, cystitis, prostate cancer, bowel cancer and haematuria) or would use such tests in the future (Yes, No, Don't Know response categories). Non-responders received one reminder.

Data analysis was undertaken using SPSS version 12 and Mintab. Analyses aimed to scope the extent and patterns of current cancer-related self-test use and produce profiles of the people who had used or would use self-testing for cancer. Participants were classified according to their use of self-test kits related to cancer (yes, no). Categorical analyses compared those reporting using self-tests with all other categories (no, don't know and blank).

Participation comprised the completion of a three page postal questionnaire. We kept questionnaire length to a minimum to maximise compliance and minimise selection bias.

Prevalence rates were directly standardised, by age, sex and deprivation, to the England population. The Index of Multiple Deprivation 2004 (IMD 2004) [28] was used as a proxy measure of multiple deprivation based on the respondent's postcode of residence. This model of multiple deprivation is based on the idea of distinct dimensions of deprivation (employment, health deprivation and disability, education skills and training, barriers to housing and services, crime and living environment), experienced by individuals living in an area, which can be recognized and measured separately. The IMD score is a weighted area level aggregation of these dimensions of deprivation. Lower IMD scores indicate less deprived areas of residence. Ranked data were converted to quartiles for analysis; quartile 1 representing the most affluent group and quartile 4 the most deprived [28].

Logistic regression was used to establish a set of characteristics which jointly distinguish those who had used a self test kit from those who had not done so. The same technique was used to distinguish those who believed that they may make use of a particular self test kit in the future from those who did not think that they would do so.

## Results

**Figure 1:**
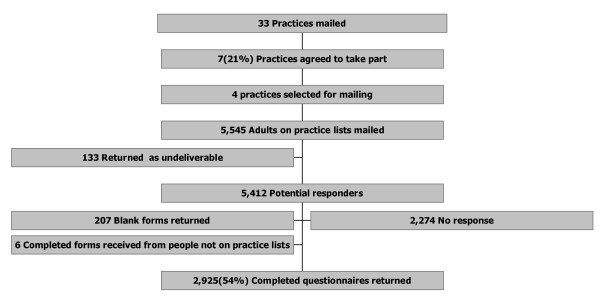
Study schematic.

Three hundred and ninety three patients were excluded from practice lists because their GPs felt it was inappropriate to send them a questionnaire. The reasons for exclusion were given as: 101 because of severe or terminal illness (including mental illness), 9 because they were practice staff, 170 because they were too frail, and 113 for other reasons. Five thousand five hundred and forty five questionnaires were mailed, but 133 were returned as "address unknown", giving a denominator of 5,412 (Figure 1). Completed questionnaires were returned by 2,925 patients and 207 were returned blank (response rate 58%, usable responses 54%).

The mean age of responders was 53 years (range 18 to 95); 45% (n = 1,308 of 2,925) were male; 92% (n = 2,698) were white; 20% (n = 578) were smokers; 88% (n = 2,580) reported their health to be good or fairly good; 51% (n = 1,495) were in paid employment and 33% (n = 970) retired; and 19% (n = 551) had a university degree (Table 1). Responders were more likely to be female (χ^2 ^= 76.1, P < 0.0001), older (χ^2 ^= 354.49 (2 DF), P < 0.0001) and from more affluent areas (χ^2 ^= 121.08 (3 DF), P < 0.0001).

**Table 1:** Baseline characteristics of participants

**Characteristics**	**All (n = 2,925)**	**Cancer self-test users (n = 35)**	Test*
**Age, years**			
Mean (range)	53.1 (18–95)	58.8 (30–87)	t=-1.92, 2923df
Median	53	60	p = 0.055
**Gende**r	N (%)	N (%)	
Male	1308 (44.7)	21 (60.0)	χ^2 ^= 3.35,1df
Female	1617 (55.3)	14 (40.0)	p = 0.067
**Index of Multiple Deprivation **(n = 2,922)	N (%)	N (%)	
Quartile 1 (least deprived)	433 (14.8)	4 (11.4)	χ^2 ^= 0.35,3df
Quartile 2	566 (19.4)	7 (20.0)	p = 0.951
Quartile 3	1230 (42.1)	15 (42.9)	
Quartile 4 (most deprived)	693 (23.7)	9 (25.7)	
**Smoking Status**	N (%)	N (%)	
Smoker	578 (19.8)	5 (14.3)	χ^2 ^= 0.85,2df
Non-smoker	2334 (79.8)	30 (85.7)	p = 0.654
Not known	13 (0.4)	0 (0.0)	
**Ethnic Group**	N (%)	N (%)	
White	2698 (92.2)	31 (88.6)	χ^2 ^= 3.71,5df
Mixed	25 (0.9)	0 (0.0)	p = 0.592
Asian	99 (3.4)	1 (2.9)	
Black	35 (1.2)	1 (2.9)	
Chinese	9 (0.3)	0 (0.0)	
Other & Not known	59 (2.0)	2 (5.7)	
**Exercise Regularly**	N (%)	N (%)	
Yes	1380 (47.2)	16 (45.7)	χ^2 ^= 0.03,2df
No	1466 (50.1)	18 (51.4)	p = 0.984
Not known	79 (2.7)	1 (2.9)	
**Health Status**	N (%)	N (%)	
Not good	324 (11.1)	5 (14.3)	χ^2 ^= 2.92,3df
Fairly good	1220 (41.7)	15 (42.9)	p = 0.404
Good	1360 (46.5)	14 (40.0)	
Not known	21 (0.7)	1 (2.9)	
**Long Term Illness**	N (%)	N (%)	
Yes	811 (27.7)	12 (34.3)	χ^2 ^= 0.76,2df
No	2020 (69.1)	22 (62.9)	p = 0.684
Not known	94 (3.2)	1 (2.9)	
**Education (highest level)**	N (%)	N (%)	
Degree	551 (18.8)	5 (14.3)	χ^2 ^= 4.50,5df
Further qualifications	1056 (36.1)	15 (42.9)	p = 0.480
Left school some qualifications	491 (16.8)	4 (11.4)	
Left school no qualifications	718 (24.5)	10 (28.6)	
Did not go to school	24 (0.8)	1 (2.9)	
Other & Not known	85 (2.9)	0 (0.0)	
**Employment**	N (%)	N (%)	
In paid employment	1291 (44.1)	11 (31.4)	χ^2 ^= 8.00,7df
Self-employed/freelance	204 (7.0)	3 (8.6)	p = 0.333
Unemployed	90 (3.1)	0 (0.0)	
Retired	970 (33.2)	16 (45.7)	
Student	63 (2.2)	0 (0.0)	
Looking after home/family	157 (5.4)	4 (11.4)	
Sick/disabled	103 (3.5)	1 (2.9)	
Other	47 (0.6)	0 (0.0)	

*Comparisons relate to cancer self-test users versus all others (i.e. non-cancer self test users and non-self test users)

Almost a third of the responders (969 of 2,925, 33.1%, 95% CI 31.4% to 34.8%) had used a self test, including pregnancy tests (19%) and blood pressure monitors (9.4%), and 287 people reported having used more than one test (Table 2). Tests for diabetes had been used by 7.7% of respondents, fertility tests by 2.4% and urinary infection tests by 2.3%. All other tests were used by less than 2% of respondents.

**Table 2:** Reported use, and potential future use, of any self-tests

	**Used**		**Would Use**	
**Test kit**	**n**	**%**	**n**	**%**
Allergy	43	1.5	1137	38.9
Cholesterol	56	1.9	1453	49.7
Chlamydia	4	0.1	504	17.2
Diabetes	225	7.7	1371	46.9
Fertility	69	2.4	496	17.0
Helicobacter pylori	28	1.0	789	27.0
Hepatitis	3	0.1	644	22.0
High blood pressure	276	9.4	1469	50.2
HIV/AIDS	2	0.1	456	15.6
Menopause	16	0.5	537	18.4
Pregnancy	555	19.0	591	20.2
Thyroid	16	0.5	753	25.7
Urinary infection	67	2.3	1027	35.1
Vaginal infection	13	0.4	612	20.9
Other	5	0.2	41	1.4
All non-cancer	1378		11880	
**Reported use, and potential future use, of cancer related self-tests**				
Bowel Cancer	8	0.3	844	28.9
Haematuria	13	0.4	690	23.6
PSA	16	0.5	639	21.8
All cancer related	*37		2173	

*37 tests used by 35 people

Thirty five people (1.2%, 95% CI = 0.83% to 1.66%) reported having used a cancer related self test kit: 8 had used a faecal occult blood test (FOB) (0.3%), 13 had used a test for haematuria (0.4%), and 16 had used a PSA test (0.5% or 1.1% of males). Two people had used two cancer related tests: one person had used PSA and FOB kits, and the other had used FOB and haematuria kits. Cancer related self-tests were used by 3.6% (35 of 969) of those who had ever used a self-test, but a further 36% (1,055 of 2,925) of respondents who had never used a cancer-related self-test reported that they would consider using one in the future. Crude and standardized rates by test, age and deprivation quartile are reported in Table 3; the highest rate of cancer-related self-testing was observed for PSA testing, rates substantially increasing with increasing age.

**Table 3:** Prevalence rates per 1,000 population – Crude and standardised to England

	Crude rate			Standardised rate		Standardised rate			Standardised rate			
Test Kit	Female (1617)	Male (1308)	All (95% CI)	Female (1617)	Male (1308)	Age <40 (747)	Age 40 – 59 (1066)	Age 60 + (1112)	IMD^1 ^Q1 (433)	IMD Q2 (566)	IMD Q3 (1230)	IMD Q4 (693)
Bowel Cancer (n = 8)	3.1	2.3	2.7 (1.2, 5.4)	3.4	1.9	1.9	1.39	5.3	4.6	3.0	1.7	1.3
Haematuria (n = 13)	5.6	3.1	4.4 (2.4, 7.6)	3.9	3.0	4.8	2.8	2.3	0	1.3	8.8	3.8
PSA (n = 16)	0.6	11.5	5.5 (3.1, 8.9)	1.1	9.3	1.0	6.3	9.4	5.3	6.3	2.8	5.7
Any cancer self-test (n = 35)	8.7	16.06	12.0 (8.3, 16.6)	8.1	13.5	7.7	10.5	15.4	9.8	9.4	12.8	10.8

^1^IMD = Index of Multiple Deprivation, 2004

The mean age of responders who had used a cancer related test was 59 years (range 30 to 87); 60% (n = 21 of the 35 cancer self-test users) were male; 89% (n = 31) were white; 14% (n = 5) were smokers; 83% (n = 29) reported their health to be good or fairly good; 40% (n = 14) were in paid employment and 46% (n = 16) were retired; and 14% (n = 5) had a university degree. Previous use of a PSA self-test was associated with increasing age (χ^2 ^= 6.43, df = 1, p = 0.01) and white ethnic group (χ^2 ^= 6.68, df = 1, p < 0.01). No significant associations were observed for haematuria or colorectal cancer although the numbers available for analysis were very small. Characteristics of cancer-related (i.e. PSA, haematuria and bowel) self-test users and non users are compared in Table 1: no statistically significant differences were observed between the two groups.

Logistic regression analyses indicated that the predictors of PSA test kit use were male gender (p < 0.01), white ethnicity (p < 0.01) older age (p < 0.01) and IMD quartile (p < 0.05); there was a lower usage in subjects from IMD quartile 3 (relatively deprived). For the haematuria test kit, looking after the home/family employment and IMD quartile 3 were both significant predictors of usage (p < 0.01 and p < 0.05 respectively). For use of a bowel cancer test there were insufficient users to make any significant conclusions although when usage was amalgamated with that for the haematuria test kit, looking after home/family again proved a significant factor (p < 0.05).

Future use of a PSA self test kit was associated with, male gender (p < 0.001), younger age (p < 0.001) and IMD quartile (p < 0.05), with men resident in IMD quartiles 1 and 2 (less deprived) being more likely to report possible future use. Future use of a haematuria test was associated with male gender (p < 0.05) and younger age (p < 0.001). For a bowel cancer self test, again future use was associated with male gender (p < 0.05), younger age (p < 0.001), and being in IMD quartiles 1 and 2 (p < 0.001)

## Discussion and Conclusion

This postal population survey found that around one in 100 of the population have used a self test for cancer and about a third of the population have used a self test for a condition other than cancer, with pregnancy tests being the most commonly used form of self-test. The two most commonly used cancer self-tests were for PSA and haematuria, even though neither PSA testing nor testing for haematuria are recommended screening procedures for cancer in the UK.

More than a third of the population (36%) report that they would consider using a cancer related self-test in the future, but it is not anticipated that all those who may consider future use would necessarily do so; actions being likely to be prompted by the onset of symptoms, experiences (e.g. diagnoses of cancer in friends or relatives) and the perceived acceptability of cancer self-testing. Similarly, if a wider range of self tests become available and their use was more widely promoted and accepted, it is possible that some of those who would not currently consider self testing may subsequently do so.

Self-testing is marketed as a valuable way of alerting people to serious health problems so that they can seek medical help [29]. The only previous UK surveys addressing the issue of self-testing report that 18% and 25% of people would, respectively, prefer self-testing to testing by a doctor and a pharmacist [30], and that 32% of people had bought a self-test kit (although this did include pregnancy tests) [31], similar to our results. Increased availability and utilisation of self-testing may have public health implications, particularly relating to the delivery of national screening programmes [32]. Although other work is now ongoing [33], this is the first study to report the prevalence of self-testing for cancer.

This study had a response rate of 54%, and it is possible that selection bias may have occurred so that responders were different from the rest of the population in terms of the frequency with which they have used self-tests. Comparison of the study population as a whole with census data for the West Midlands suggests that respondents were older (mean age: census 42, study 54) and more likely to be female (census 51%, study 55%) but that the study population included similar proportions of people describing themselves as having white ethnicity (census 91%, study 92%). Standardised rates were calculated to allow for the effect of response bias.

It is possible that respondents may have misinterpreted the question about self-test use and may have included the use of home monitoring, as opposed to self-diagnosis. However this is unlikely to have affected the estimate of cancer related test use, as such tests are not used for self-monitoring. Conversely, it is possible that some respondents perceived self-testing to be an unacceptable activity and social desirability bias may have resulted in the reported rate of use underestimating the true rate. We have no information at present to determine the accuracy of the responses to the survey. However, an ongoing multi-method study [33] is considering respondent's understanding of the definition of self-testing and the factors associated with use; this work will provide further information relating to these potential biases.

We have demonstrated that 1% of the adult population reported having used a cancer-related self-test kit and that more than a third of the adult population reported that they would consider using such a test in the future. Cancer related self-testing may develop to include tests for the early diagnosis of cancers at more sites, the genetic determinants of disease [21] and drug effectiveness [22]. Self-tests for cancer could alter perceptions of risk and health behaviour, cause psychological morbidity, could potentially be misused (e.g. if testing became a precedent to employment) and could have a significant impact on the demand for healthcare.

## Competing interests

The author(s) declare that they have no competing interests.

## Authors' contributions

HP, SG, SW, AR, DF and RM had the idea for the study, AR and SC were responsible for data collection, RH supervised statistical aspects and undertook the regression analyses. All authors contributed to the design of the study and interpretation of the data. SW wrote the first draft of the manuscript with significant input from all authors. All authors have read and approved the final manuscript.

## Pre-publication history

The pre-publication history for this paper can be accessed here:


